# An update on renal involvement in hemophagocytic syndrome (macrophage activation syndrome)

**DOI:** 10.15171/jnp.2016.02

**Published:** 2015-07-15

**Authors:** Haydarali Esmaili, Elmira Mostafidi, Bahareh Mehramuz, Mohammadreza Ardalan, Mohammadali Mohajel-Shoja

**Affiliations:** ^1^Department of Pathology, Al-Zahra Hospital, Tabriz University of Medical Sciences, Tabriz, Iran; ^2^Kidney Research Center, Tabriz University of Medical Sciences, Tabriz, Iran; ^3^Pediatric Neurosurgery Unit, University of Alabama, Birmingham, USA

**Keywords:** Hemophagocytic syndrome, Macrophage activation syndrome, Interferon-gamma, Thrombotic microangiopathy

## Abstract

*Context:* Hemophagocytic syndrome (HPS) is mainly characterized by massive infiltration of bone marrow by activated macrophages and often presents with pancytopenia. Thrombotic microangiopathy (TMA) is also present with thrombocytopenia and renal involvement. Both conditions could coexist with each other and complicate the condition.

*Evidence Acquisition:* Directory of Open Access Journals (DOAJ), EMBASE, Google Scholar, PubMed, EBSCO, and Web of Science with keywords relevant to; Hemophagocytic syndrome, macrophage activation syndrome, interferon-gamma and thrombotic microangiopathy, have been searched.

*Results:* Viral infection, rheumatologic disease and malignancies are the main underlying causes for secondary HPS. calcineurin inhibitors and viral infections are also the main underlying causes of TMA in transplant recipients. In this review, we discussed a 39-year-old male who presented with pancytopenia and renal allograft dysfunction. With the diagnosis of HPS induced TMA his renal condition and pancytopenia improved after receiving intravenous immunoglobulin (IVIG) and plasmapheresis therapy.

*Conclusions:* HPS is an increasingly recognized disorder in the realm of different medical specialties. Renal involvement complicates the clinical picture of the disease, and this condition even is more complex in renal transplant recipients. We should consider the possibility of HPS in any renal transplant recipient with pancytopenia and allograft dysfunction. The combination of HPS with TMA future increases the complexity of the situation.

**Implication for health policy/practice/research/medical education:**

Hemophagocytic syndrome (HSP) (macrophage activation syndrome) is an increasingly recognized disorder in the realm of different medical specialties. Renal involvement complicates the clinical picture of the disease, and this condition even is more complex in renal transplant recipients. We should consider the possibility of HPS in any renal transplant recipient with pancytopenia and allograft dysfunction.

## 1. Context

Hemophagocytic syndrome (HPS) also known as macrophage activation syndrome or hemophagocytic lymphohistiocytosis (HLH), is a massive infiltration of bone marrow and lymphoid organ by activated macrophages and often presents with fever, pancytopenia, adenopathies, hepatosplenomegaly, hyperferritinemia and hypertriglyceridemia (1). Clinical signs are variable, nonspecific and often evolves in a subacute course within 1-4 weeks (2). Primary HPS occurs in children with inherited dysfunction of the immune response, particularly natural killer (NK) cells and cytotoxic T-cell the condition subsequently leads to cytokine elevation and mononuclear-phagocytes activation (2,3). Secondary HPS is often triggered by infectious, autoimmune, and neoplastic diseases, and sometimes more than one underlying cause dose exist (2).

## 2. Evidence Acquisition

For this review, we used a variety of sources by searching through PubMed/Medline, Scopus, EMBASE, EBSCO and directory of open access journals (DOAJ). The search was conducted, for articles published from January 1, 1970 up to September 29, 2011, using combinations of the following key words and or their equivalents; Hemophagocytic syndrome, macrophage activation syndrome, interferon-gamma and thrombotic microangiopathy.

## 3. Mechanisms and diagnostic criteria

HPS’s staring point is the inability of immune system to control the primary causative infectious lead to hyper-secretion of pro-inﬂammatory cytokines including; Tumor necrosis factor-alpha (TNF-a), interferon-gamma (IFN-γ). Interleukin 1, interleukin 4, interleukin 6, interleukin 8, interleukin 10, interleukin 18, and creating a condition that is named cytokine storm (4-6). Fever, cachexia, elevated serum triglyceride, high serum ferritin level, acute kidney injury (AKI) and tubular necrosis that are present in this situation all are attributed to high cytokines levels (1,4).

Diagnosis of HPS or HLH is based on the presence of at least 5 or more of the following conditions; fever >38.3°C, splenomegaly, pancytopenia that defines two of the followings (hemoglobin <90 g/dl, platelets <100 000/μl, and neutrophils <1000/μl), hypertriglyceridemia and/or hypoﬁbrinogenemia and hemophagocytosis diagnosed in bone marrow, spleen, or lymph nodes biopsy. Immunologic markers include low or absent NK-cell activity (assessed by chromium 51 or granzyme B proteolytic activity). Key diagnostic immunologic markers are increased plasma level of CD163, as a marker of macrophage activation, and CD25 as an interleukin 2 receptor (sIL-2R), is increased is in 79% of adult with HPS (7,8). The bone marrow aspiration is the best investigation site and identifies mature histiocytes ingesting other blood cells in 84% of conditions (9). Liver is another frequently involved organ and 60% of patients have abnormal liver tests. Splenic involvement and rupture, ascites, veno-occlusive disease, pulmonary involvement and encephalopathy are other rare reported conditions in HPS. Renal involvement in HPS will be discussed later and it is a predictor of poor prognosis (10,11).

### 3.1. Hemophagocytic syndrome in adults

Secondary HPS was first described at 1979 in 19 patients interestingly, thirteen of of these patients were renal transplant recipients. In transplant recipients, HPS often triggers by an infection, mainly viral, including adenovirus, human herpes virus 8 (HHV-8), Epstein-Barr virus (EBV), cytomegalovirus (CMV) (12,13), HHV-6 (14), parvovirus B19 (15,16) and BK polyomavirus (17), herpes viruses account for 62% of viral cases of HPS, and EBV is known as the most prominent of them (2). Tuberculosis, toxoplasmosis, leishmaniosis, babesiosis, Rickettsia spp, and Staphylococcus spp and* Escherichia coli* have been reported as the causative infections triger for initiation and development of HPS (18-20)*.* Among the different features of tuberculosis the role of extra pulmonary tuberculosis is more prominent (21). *Pneumocystis jiroveci*, *Toxoplasma gondii*, and fungi infection are other possible triggers (2). HPS has been reported in chronic hepatitis B or hepatitis C virus infections (22). In addition, lymphoma and sarcoma should also be considered as another possible trigeres in transplant recipients. T-cell lymphoma is related to EBV infection and metacentric Castleman disease is also associated with herpesvirus-8 infection interestingly both of these conditions could be trigeres for HPS development. In rheumatologic disorders associated-HPS, such as systemic lupus erythematous (SLE), adult-onset Still’s, systemic vasculitis, and inﬂammatory bowel disease, also, various infections are the main underlying trigger (2,23). Excessive cytokine secretion by tumor cells could be the main trigger in malignancy associated HPS (2,24,25).

**Figure 1:**
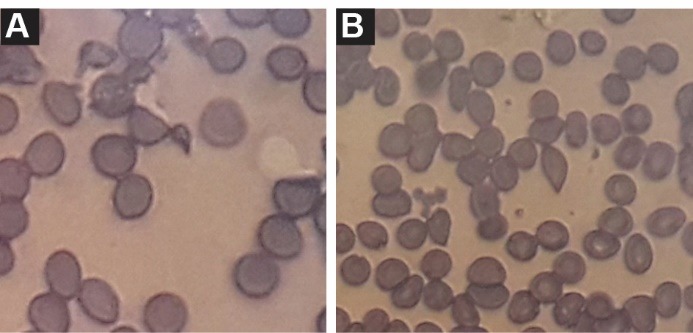
Peripheral blood smear (10*40) shows helmet cells (schistocytes) in the central lesions of both slide field that is compatible with thrombotic microangiopathy.

**Figure 2:**
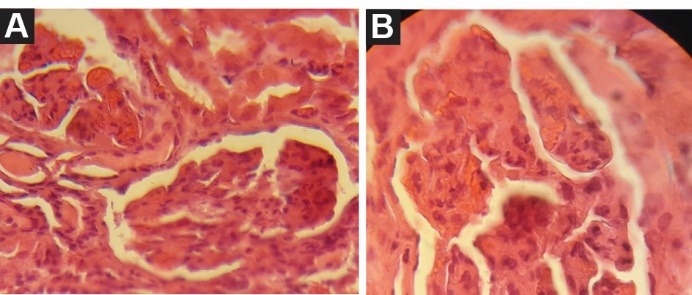
Renal biopsy , A) (40*10 ) and B) (100*10), glomerular capillary loops are constricted with fibrin thrombi and endothelial swelling, without any cellular infiltration.

Here we describe a renal transplant recipient with HPS and TMA with renal involvement. A 39-year-old male was admitted for a 2-week onset of fever, weakness and anemia. He received a second living unrelated renal transplantation 9 months ago and was on triple immunosuppressive therapy with tacrolimus (3 mg/day), mycophenolate mofetil (1.5 mg/day) and prednisolone (5 mg/day). He received multiple sessions of plasma exchange, and intravenous immunoglobulin (IVIG) before transplantation and induction therapy with rituximab, basiliximab, and anti-thymocyte globulin (ATG). One month before admission, his serum creatinine level was 1.2 mg/dl, however it was raised to 3.3 mg/ dl in the next admission. Physical examination revealed a blood pressure of 90/70 mm Hg, body temperature 38.5°C and patient was alert. Hepatosplenomegaly or lymphadenopathy was not detected. Laboratory examination revealed hemoglobin; 6 mg/dl, white blood cell; 19 000/μl (60% polymorphonuclear cells), platelets count; 35 000/μl, normal prothrombin (PT) and partial thromboplastin (PTT) times. Peripheral blood smear showed high proportion of schistocytes (Figure 1). Reticulocyte count was 2.4%, ﬁbrinogen level; 3.8 mg/dl (2–4 mg/dl) and level of D-dimer was in normal range, serum lactate dehydrogenase (LDH); 850 (105–333 IU/l), serum ferritin; 2634 (12–150 ng/ml), triglyceride; 8100 mg/dl. Urine dipstick proteinuria was trace. A 24-hour urine protein excretion was 300 mg/day. Renal allograft ultrasonography disclosed a normal size and texture. Workup for infective etiology, including urinary tract infection and pulmonary infection was negative. Serologic study for CMV, EBV and parvovirus B19 was negative. The serum PCR examinations were also negative for above infections. Furthermore, serologic study for toxoplasmosis was also negative. Bone marrow aspiration revealed an increase in histiocytes with widespread hemophagocytosis. With the diagnosis of TMA and HPS, patient received IVIG, and 2 liter/day therapeutic plasma exchange (TPE) with fresh frozen plasma replacement. After eight cycles, patient’s condition improved clinically. LDH decreased and platelet increased to 120 000/μl. At this time a renal biopsy was conducted. In light microscopy, we found endothelial cell swelling resulted in glomerular capillary narrowing and obliteration. In some parts the capillary lumen was occluded by fibrin thrombi which was indicative of TMA (Figure 2). The disease course stabilized gradually. Patient discharged with clinical and laboratory improvement and good general condition. The last serum creatinine was 1.9 mg/dl. This case history is an example of difficulties on diagnosis and therapeutic options of these patients.

Renal transplant recipients are very prone to HPS. Their immunosuppression and deregulated immune system are risk factors for both opportunistic infections and malignancies. Despite this situation, HPS is a rare condition. However, it is possible that this syndrome is under diagnosed among this population. Sign and symptoms of HPS easily overlooked by the signs and symptoms of underlying infection. Post-transplant HPS usually happens during the early period when patients are in highest immunosuppressed condition. Polyclonal antithymocyte antibodies and history of acute rejection treatment are predisposing conditions (26,27). The underlying viral, bacterial, and protozoa infections are the main contributor contributors (28). Posttransplantation lymphoproliferative disorders and solid tumors should be considered as important underlying causes (28).

There is a high mortality rate among renal transplant recipients with HPS (26-46). In a cohort study conducted by Karras et al (28) 8 of 17 renal transplant recipients died and 4 of the 9 survivors lost the allograft. However, underlying infectious disease, malignancies, immunosuppressive regimen side effects and liver involvement could be the risk factors of HPS associated mortality and morbidity among these patients (25). Diﬀerential diagnosis of the HPS from a severe presentation of the underlying infection, malignancy or autoimmune disease is an important diagnostic challenge (29). Neurological and psychiatric presentations of HPS happen in one fourth of patients. They are heterogeneous and include coma, seizures, meningitis, cerebral hemorrhage, mood disorders, delirium or psychosis (2,23). In renal recipient patients with unexplained fever and neurological symptoms, the possibility of toxoplasmosis should also be considered. Interestingly, the toxoplasma infection by itself could be a triger for HPS development and this combination creates diagnostic difficulties (2).

Management of HPS in renal transplant recipients requires immunosuppressive dose reduction and introduction of specific antiviral treatment. High-dose polyvalent immunoglobulin is a beneficial treatment (30). These patients often receive steroids and cyclosporine as their immunosuppression regimen, as this regimens also has been proposed for treatment of HPS. However, the mortality of HPS in renal transplant recipients is high (30).

Interestingly, the highest frequency of HPS occurs in umbilical cord blood derived hematopoietic stem cell transplantation (HSC) and it happens in 17% of the cases. In kidney transplanted patients, HPS happens only in 0.4%–2% of recipients (2). Allograft rejection by itself could be a trigger for HPS evolution (2). Despite profound workup, some patients with HPS remains underdiagnosed (2). Diagnosis of post-transplant HPS is not easy, pancytopenia may be caused by immunosuppressive drugs, and hypertriglyceridemia is frequent in these patients, particularly, in those receiving high doses of steroids, sirolimus or everolimus. High levels of serum ferritin level is helpful, however, its elevation could be detected in other inflammatory conditions (26,30).

### 3.2. Renal involvement in HPS

AKI is the most common renal disorder in HPS followed by nephrotic syndrome. In general, the prognosis of HPS related renal involvement is not good. AKI in HPS could be as a part of the multiorgan failure due to increased capillary permeability and pre-renal condition. It can also be related to the nephrotoxic effects of antimicrobial therapy. Acute tubular necrosis is often associated with interstitial inflammation. Glomerular involvement is less common in HPS. Nephrotic syndrome could be due to minimal-change disease or collapsing glomerulopathy. TMA renal involvement in HPS is a very rare condition (31-33).

Tubular destruction is the result of inflammatory cytokines damage (34-36). The serum level of tumor necrosis factor α (TNFα) in HPS patients is very high, and might have some roles in tubular necrosis and glomerular involvement (37). TNFα band to its receptor (TNFR1) mediates renal damage through increasing granulocyte infiltration and activation of apoptotic signaling kinase-1 (ASK1) in tubular cells (38,39). The idea of pro-inflammatory cytokines renal epithelial damage is supported by the clinical observation of AKI in the absence of circulatory disorders, referred to the direct tubular toxic effect of high serum levels of IL-6 and TNFα (35,40,41). TNFα can disorganize the podocytes actin cytoskeleton leads to increase glomerular permeability to albumin. Genetic background affects the degree and type of podocyte lesion in this condition (16). HPS-associated glomerulopathy is a rare condition and often is associated with sever nephrotic syndrome combined with renal dysfunction that may need dialysis during the course of HPS. Collapsing glomerulopathy was the most common pathologic feature that only was observed among black patients and minimal-change disease was the second common finding (40,41). TNFα and TNFRs are also important determinants of renal transplant rejection. Expression of TNFRs is up-regulated in normal kidney, and based on the type of receptor predeominancy (i.e. TNFR1 or TNFR2) inflammation progresses or halts (39,42,43). Collapsing glomerulopathy and nephrotic syndrome are less common than tubular damage. However, it is very important diagnosis in HPS related renal involvement. The development of proteinuria is correlated with the systemic and organ-specific signs and cytokine burst. Glomerular involvement is not related to immune deposits, rather, it is a podocyte injury because of actin cytoskeleton degeneration and genetic background also influence the condition. Interestingly serum TNFα in 3 patients with HPS and glomerulopathy was more than 10 times the maximal normal value (28,31,44).

TMA is another type of glomerular involvement in HPS (2,7). TMA is a more general term for both thrombotic thrombocytopenic purpura (TTP) and hemolytic uremic syndrome (HUS). It is a multisystem disorder characterized by consumptive microangiopathic hemolytic anemia, thrombocytopenia, with various degrees of neurologic symptoms, and renal impairment. Simultaneous existence of HPS and TMA has been confirmed in a few reports (15,45). Renal involvement in TMA could be the consequence of cytokine induced endothelial injury (15,45). There is also a reported case of HPS triggered by plasma exchange that was started for treatment of TTP, and the condition was explained by high amounts of cytokines released from neutrophils during their passage to the plasma exchange materials (46,47). Furthermore, there are two reported cases of HPS-associated with preeclampsia. Interestingly, the pathologic feature of the glomerular endotheliosis and capillary loop occlusion in preeclampsia are very similar to the renal lesions by TMA (33,47).

### 3.3. Therapeutic options

Elimination of triggers (mainly infections) is crucial for treatment of adult patients with HPS. High-dose polyvalent IVIG is beneficial in infection, autoimmune, and transplant related HPS (48). The main predictive factor of response is its early administration particularly when ferritin level is high (49,50). C-reactive protein, erythrocyte sedimentation rate (ESR), improvement of hypertriglyceridemia, and hyperferritinemia may be useful indicators of disease activity. Steroids and cyclosporine have been proposed as a treatment for HPS (31,50). Biological treatments such as rituximab, inﬂiximab, and etanercept (Enbrel) have been proposed for adults patients who did not respond to cyclosporine and IVIG, (51) tacrolimus, and etoposide also have been proposed in refractory cases (52).

Use of anti-TNFα drugs, anti-IL1receptor (Anakinra) antibody, and anti-IL6 (tocilizumab) has been proposed for rheumatoid arthritis and adult Still’s induced HPS (53). B-cell depleting drugs (rituximab, belimumab) have been proposed for SLE, and EBV-related HPS with or without associated lymphoma.

Use of cyclosporine in those patients who did not respond to IVIG and alemtuzumab, has been used in three adult patients with HPS with reliable results (54,55). For treatment of HPS in allograft recipients, at first we should try to find the underlying triger and target it, and in most of the condition the triger is infection. When there is a combination of HPS and TMA addition of therapeutic plasma exchange is useful. Graft nephrectomy has been proposed for kidney transplant recipients with life-threatening HPS resistant to different therapies (53).

## 4. Conclusions

HPS is an increasingly recognized disorder in the realm of different medical specialties. Renal involvement complicates the clinical picture of the disease, and this condition even is more complex in renal transplant recipients. We should consider the possibility of HPS in any renal transplant recipient with pancytopenia and allograft dysfunction. The combination of HPS with TMA future increases the complexity of the situation. However these conditions and their combination may not be diagnosed, if unsuspected.

## Authors’ contribution

Primary draft by HE, EM, BM and MRA. Editing the final manuscript by MMS.

## Conflicts of interest

The authors declared no competing interests.

## Funding/Support

None.
